# Supplementary figures and images for: Talaromyces santanderensis: A New Cadmium-Tolerant Fungus from Cacao Soils in Colombia
Source: J Fungi (Basel). 2022 Oct 1;8(10):1042. doi: 10.3390/jof8101042 (PMC9605138; doi:10.3390/jof8101042)

# CAM

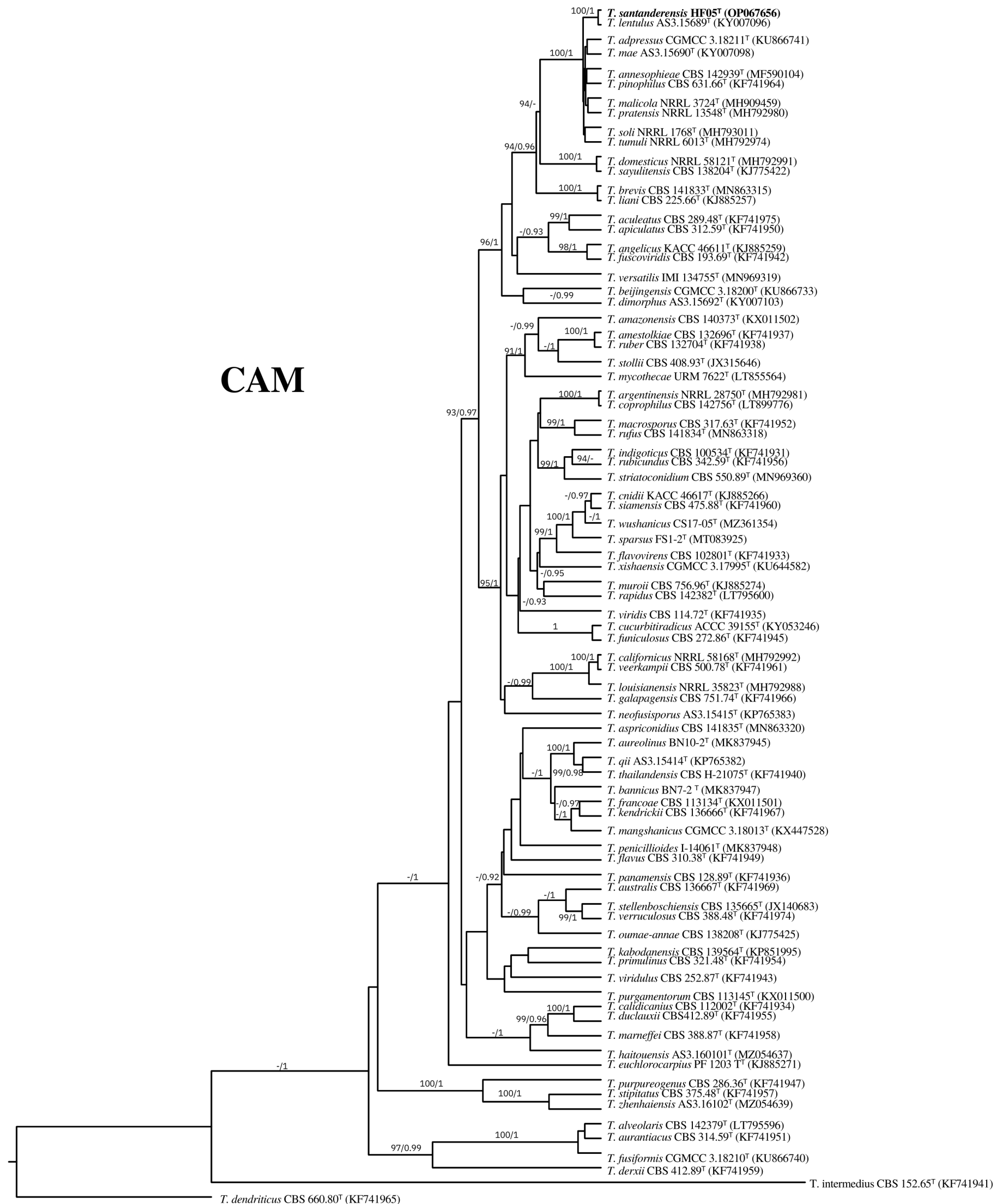

0.06

Supplement: Supplementary file 1 [file jof-08-01042-s001.zip › Figure S1.pdf]

RPB2

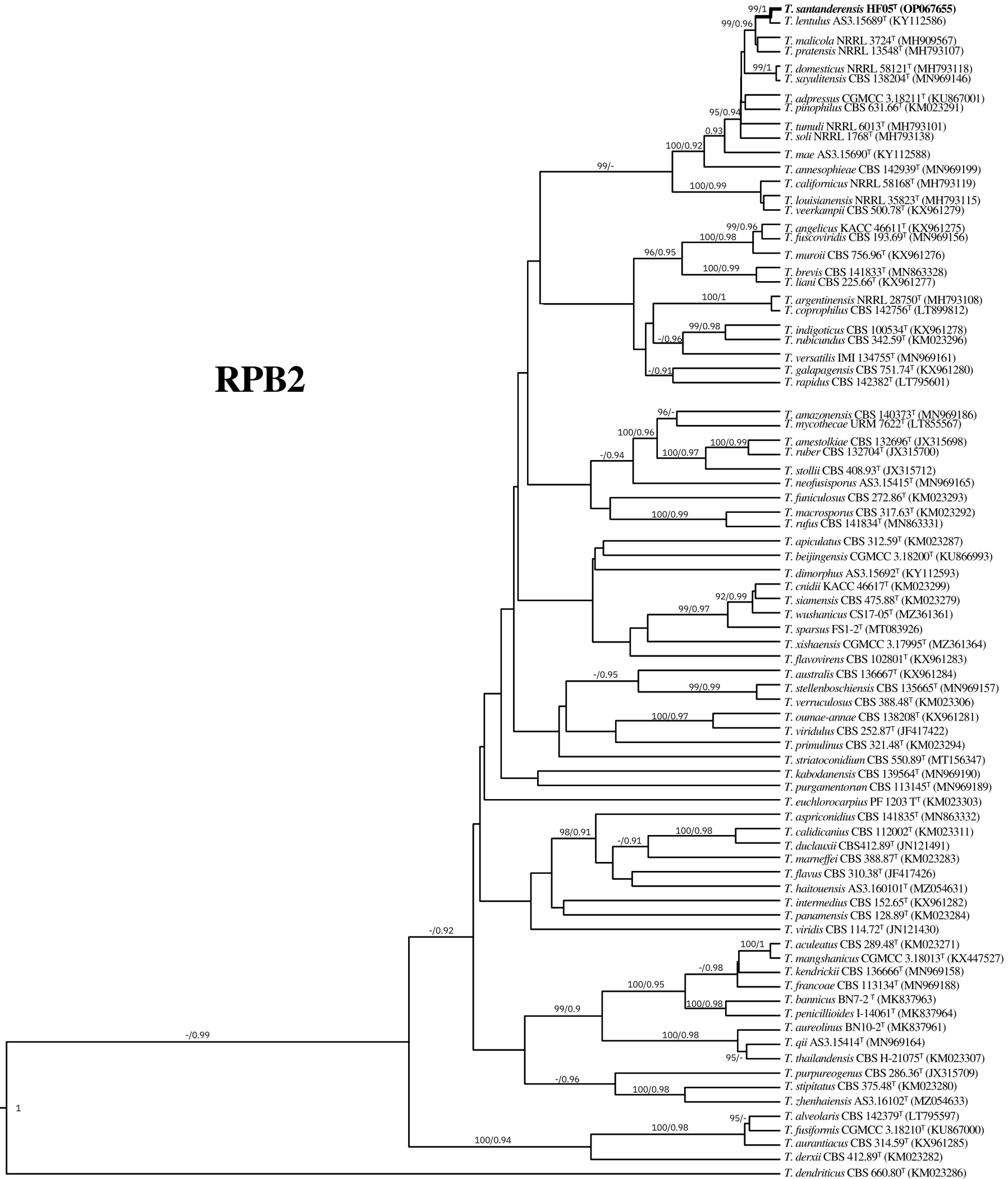

Supplement: Supplementary file 1 [file jof-08-01042-s001.zip › Figure S2.pdf]

ITS

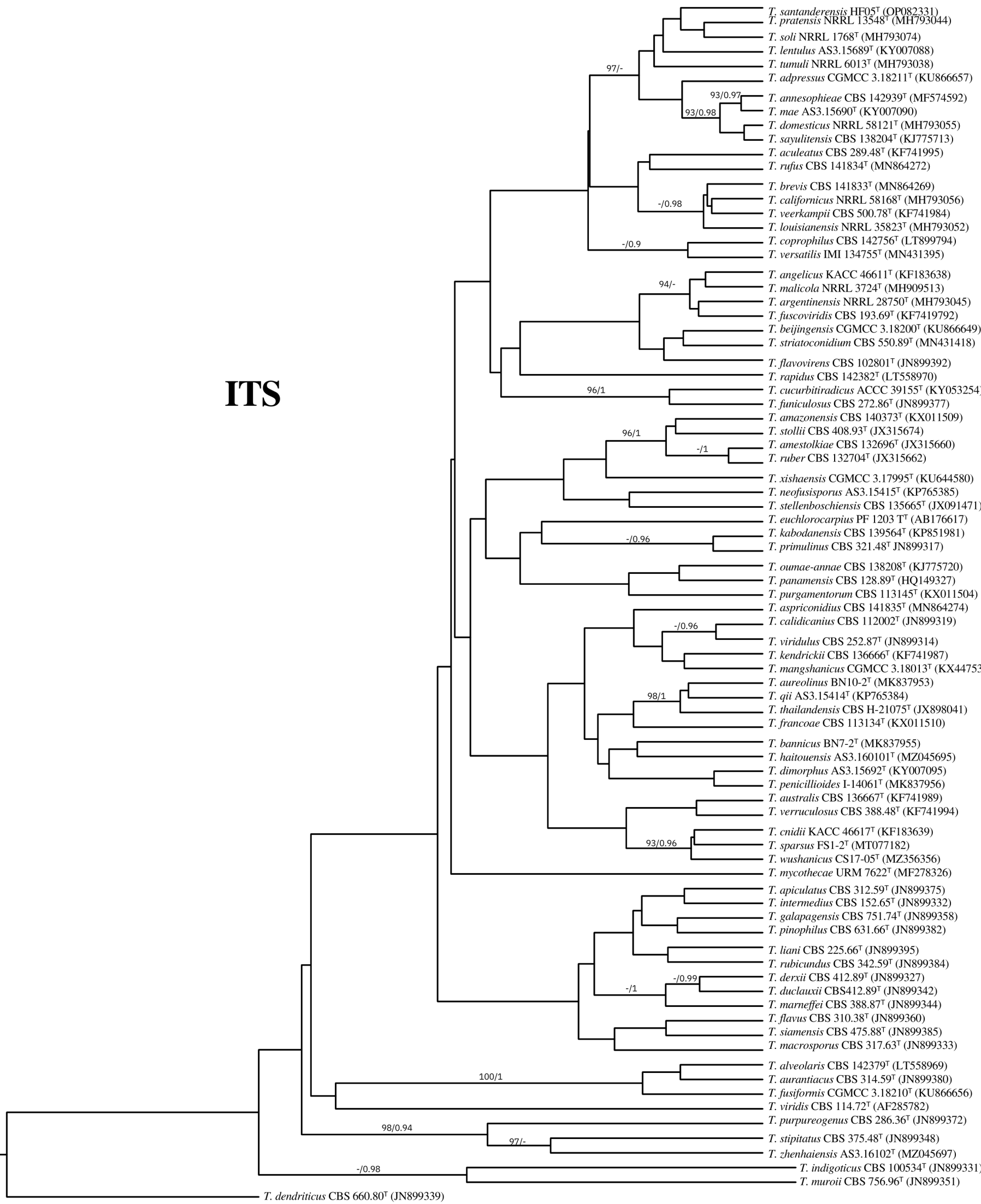

0.004

Supplement: Supplementary file 1 [file jof-08-01042-s001.zip › Figure S3.pdf]
